# What makes a megaplasmid?

**DOI:** 10.1098/rstb.2020.0472

**Published:** 2022-01-17

**Authors:** James P. J. Hall, João Botelho, Adrian Cazares, David A. Baltrus

**Affiliations:** ^1^ Department of Evolution, Ecology and Behaviour, Institute of Infection, Veterinary and Ecological Sciences, University of Liverpool, Liverpool, UK; ^2^ Antibiotic Resistance Evolution Group, Max Planck Institute for Evolutionary Biology, Plön, Germany; ^3^ Department of Evolutionary Ecology and Genetics, Zoological Institute, Christian Albrechts University, Kiel, Germany; ^4^ EMBL's European Bioinformatics Institute (EMBL-EBI), Wellcome Genome Campus, Cambridge, UK; ^5^ Wellcome Sanger Institute, Wellcome Genome Campus, Cambridge, UK; ^6^ School of Plant Sciences, University of Arizona, Tucson, AZ, USA

**Keywords:** plasmid, megaplasmid, mobile genetic element, genome evolution, horizontal gene transfer, pangenome

## Abstract

Naturally occurring plasmids come in different sizes. The smallest are less than a kilobase of DNA, while the largest can be over three orders of magnitude larger. Historically, research has tended to focus on smaller plasmids that are usually easier to isolate, manipulate and sequence, but with improved genome assemblies made possible by long-read sequencing, there is increased appreciation that very large plasmids—known as megaplasmids—are widespread, diverse, complex, and often encode key traits in the biology of their host microorganisms. Why are megaplasmids so big? What other features come with large plasmid size that could affect bacterial ecology and evolution? Are megaplasmids 'just' big plasmids, or do they have distinct characteristics? In this perspective, we reflect on the distribution, diversity, biology, and gene content of megaplasmids, providing an overview to these large, yet often overlooked, mobile genetic elements.

This article is part of the theme issue ‘The secret lives of microbial mobile genetic elements’.

## 1. Megaplasmids: very large plasmids

The word plasmid was coined in 1952 to refer to any extra-chromosomal ‘hereditary determinant’ [1]. Though this broad term originally included endosymbionts, in the years since it has been narrowed to refer principally to DNA extrachromosomal genetic elements that, unlike phage, do not encode capsids for transmission. Nevertheless, plasmids can be extraordinarily diverse—in terms of composition, with G + C content ranging from 20% to almost 90% [2]; in structure, with linear and circular forms, and the ability to integrate into the chromosome or excise and replicate independently [3–5]; in host range [6]; in mechanism of replication [7]; in gene content [8]; and (like the microbes that host them) in the environments in which they are found [9–12]. Natural plasmids can be found in bacteria, archaea and eukaryotes, such as yeast and slime moulds [13–15]. Perhaps the most striking manifestation of plasmid diversity is their size. Plasmid genomes span three orders of magnitude, with the largest coming in at around 2.5 Mb—3500× bigger than the smallest plasmids (which can be less than 800 bp), and approximately 4.5× the size of the chromosome of some culturable bacteria [2,16]. Plasmids can clearly be very small, and they can also be very big.

**Figure 1.**
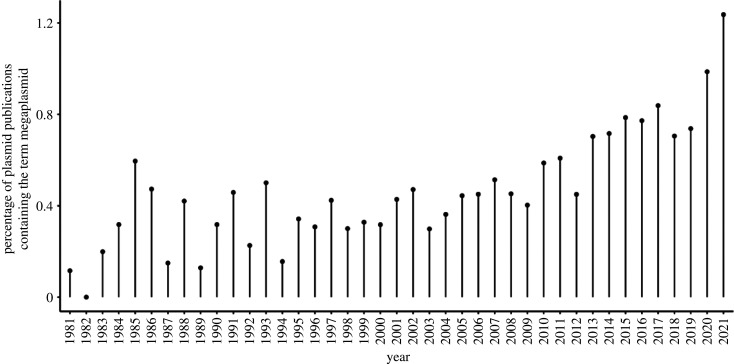
Percentage of plasmid publications containing the term ‘megaplasmid’. Metadata on publications containing the term ‘plasmid’ or ‘megaplasmid’ from 1981 to date were retrieved separately from Pubmed (https://pubmed.ncbi.nlm.nih.gov/advanced/) using the following search queries: for ‘plasmid’ - (plasmid[Title/Abstract]) AND ((‘1981’ [Date - Publication] : ‘3000’ [Date - Publication])); for ‘megaplasmid’ - (megaplasmid[Title/Abstract]) OR (mega-plasmid[Title/Abstract]) AND ((‘1981’[Date - Publication] : ‘3000’[Date - Publication])). The number of publications recorded for each term per year was used to calculate the percentages displayed in the plot.

Very large plasmids were first called ‘megaplasmids’ by Rosenberg and colleagues in the early 1980s. By employing a gentle DNA extraction process to investigate the genetics of *Sinorhizobium meliloti*, they were able to identify extrachromosomal elements that were too large and fragile to be retrieved by their previously-used alkaline lysis approach [17,18]. With an apparent molecular weight ‘clearly larger’ than 300 × 10^6^ Da—which is approximately 450 kb—they termed these elements megaplasmids. The term has persisted, and increased in popularity (figure 1) as a handle referring to very large plasmids, bounded on one side by size (i.e. a small megaplasmid is ‘just’ a plasmid), and on the other by the extent to which they are integrated into cellular physiology, with very large additional replicons that are essential for viability termed secondary chromosomes or ‘chromids’ [19–21].

It is apt that megaplasmids were initially defined operationally in the context of an extraction protocol, as megaplasmid identification in the years since has largely followed the prevailing technical norms. Many megaplasmids were first characterized during the period when microbial genetics was pursued principally through use of gel electrophoresis and hybridization [22,23]. The advent of accessible short-read sequencing technology precipitated a genomics revolution in microbiology, but while this flood of data vastly improved our understanding of microbial genome content, unambiguously resolving megaplasmids remained difficult, because megaplasmids are usually maintained at low copy number, and carry sequences which are repetitive, or similar to those in the chromosome. Assembly often failed to yield complete megaplasmid contigs, and it is likely that short-read databases hold many megaplasmids that are not annotated as such, as recently demonstrated for a family of megaplasmids identified in *Pseudomonas aeruginosa* [24]*.* The recent availability of long-read single-molecule sequencing technologies, such as the PacBio SMRT protocol and Nanopore devices, enables megaplasmids to be unambiguously resolved [25–27], and with this increased detection rate, recent years have seen a steep increase in the proportion of plasmid publications containing the term ‘megaplasmid’ (figure 1).

The dynamic, mosaic, modularity of microbial mobile genetic elements means that the borders of any category—plasmid, phage, transposon, integrative and conjugative element (ICE)—are somewhat fuzzy [28–30], and this issue poses a particular problem for megaplasmids. Are megaplasmids ‘just’ big plasmids, or is there something beyond size *per se* that sets megaplasmids apart? Is there any biological meaning in a term that was first used in relation to a DNA extraction procedure? In this perspective, we explain the problems with defining megaplasmids, and assess whether there is a distinct, coherent category of mobile genetic elements we can refer to as megaplasmids. We discuss what features of very big plasmids might distinguish them from other genetic compartments, how and why they became so large, and what they reveal about microbial evolutionary ecology. Throughout, we give some examples of how megaplasmids contribute to the biology of diverse microbes.

## 2. How big is a megaplasmid?

**Figure 2.**
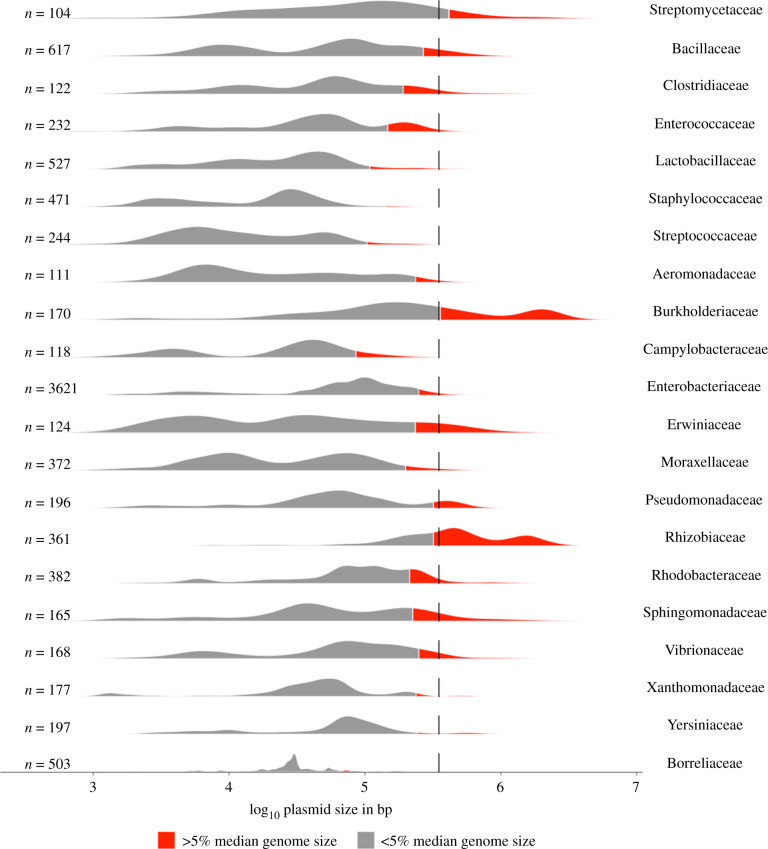
Plasmid size distributions for families with greater than 100 plasmids in the COMPASS database [13]. The number of plasmids analysed is displayed to the left of each graph. Red filled areas correspond to 5% of the median genome size for each family, according to EZBioCloud [31]. The dotted line indicates 350 kb [19]. Plots were generated in R using the ggplot and tidyverse packages. See the electronic supplementary material for further details.

If plasmid size is plotted as a histogram, most species give a bimodal distribution (figure 2). The smaller peak, at around 10^3^–10^4^ bp, corresponds to (usually) multi-copy, non-conjugative plasmids, like the ColE1 plasmids, whereas the larger peak, at around 10^4^–10^5^ bp, corresponds to low-copy, often conjugative plasmids, like RP4 [32]. This bimodality reflects a natural division, roughly separating two classes of plasmid which have distinct biological features and behaviours [33]. Rather than a distinct third peak, megaplasmids tend to appear as part of a continuous spectrum, or shoulder, on the right-hand of the larger plasmid peak. There are some exceptions: distinct ‘megaplasmid’ peaks can be seen in the distributions of plasmids from Pseudomonadaceae, Rhizobiaceae, Burkholderiaceae and Enterococcaceae, for example. These mainly appear to represent related plasmids within each family, which could reflect underlying biological differences or fitness benefits associated with large plasmid size. Overall, megaplasmids appear to share many of the general features of plasmids comprising the large plasmid peak, including replication mechanism, copy number and transmissibility, and thus do not appear distinct from large plasmids the way large plasmids do from small ones [33,34]. More sensitive assignment may be possible as more sequences are collected, and methods developed to control for phylogenetic correlation in a mosaic and recombining group of elements that lacks core conserved genes, but currently, defining megaplasmids is usually achieved through a rather unsatisfying process of slicing the pool of plasmids at an arbitrary size threshold.

Thresholds for minimum megaplasmid size have thus varied. diCenzo and Finan's seminal review on multipartite genomes set the threshold at 350 kb, based on 10% of median bacterial genome size [19], while 100 kb has been used for other studies [20], but as genome size itself varies widely across phyla, the very largest plasmids in some species might be only of middling size in others (figure 2). Fixed thresholds are likely to bias megaplasmids towards species with already large genomes, since plasmid size and genome size are correlated [33]. The ecological and evolutionary factors driving large plasmid size are likely to apply similarly to phylogenetically diverse species, so to understand the biology of very large plasmids we consider it more appropriate to regard plasmid size relative to other replicons in that species. For example, *Campylobacter* and *Aliarchobacter* plasmids of only approximately 100–150 kb can harbour greater than 7% of total genome content [35,36], which in *Pseudomonas* would be equivalent to greater than 450 kb. In figure 2, we highlight plasmids that are relatively large: greater than or equal to 5% of total genome size for that family. Nevertheless, as the figure shows, even proportional thresholds artificially divide what is in fact a continuous distribution, and so we emphasise that our use of 5% is intended to be illustrative rather than prescriptive.

Megaplasmids have also been distinguished from the other large replicons: secondary chromosomes and ‘chromids’ [21]. This difference is a qualitative one: chromids and secondary chromosomes carry essential housekeeping genes found on the chromosome in other species, often confirmed experimentally. Compared with plasmids, chromids and secondary chromosomes tend to have genomic signatures—such as GC content, codon usage and dinucleotide relative abundance—that more closely resemble those of the chromosome [19,21]. The distinction between secondary chromosomes and chromids comes down to their evolutionary history: secondary chromosomes formed by the split of an ancestral chromosome, while chromids most likely originated from megaplasmids [19]. Secondary chromosomes thus have replication machineries that are clearly distinct from those of chromids and megaplasmids [37], and on this basis it appears that secondary chromosomes are less common than other large replicons (though there are some possible natural and artificial examples [19,38]). Because carriage of essential genes is a discrete and measurable trait, distinguishing megaplasmids from chromids appears conceptually less arbitrary than distinguishing megaplasmids from large plasmids. However, determining essentiality is not always easy, and not always consistent across environments and strains [39,40]. Definitive classifications may also require the evaluation of replicons in a context that takes into account conservation through evolutionary time and over different levels of taxonomy. For instance, megaplasmids are considered to be more plastic whereas chromids and secondary chromosomes may be found more consistently in every member of a clade as a consequence of their essentiality [21]. Regardless of underlying definitions and classification schemes, it is currently apparent that ‘megaplasmid’, ‘chromid’ and ‘secondary chromosome’ are not used consistently across the literature.

Taxonomies of mobile genetic elements have been notoriously difficult owing to their mosaic structure, their diversity, their propensity to recombine, and horizontal transmission across phylogenetic boundaries [8,41–44]. For example, some ICEs are more closely related to plasmids than other ICEs, with plasmid-like features such as extrachromosomal replication and partitioning systems [45,46]. Recent network analyses have had some success in categorizing plasmids into clusters based on genome-level pairwise similarities, but such schemes ignore core functional identities of plasmids like replication and transmission, and can still produce edge cases and mosaics that defy unambiguous classification [8,47]. Overall, though there may be trends, exceptions are often the rule. Arguing over ‘what counts’ as a megaplasmid may not be helpful in understanding their biology, as replicons have the potential to cross size thresholds and transition between essentiality/non-essentiality with ease. With these concepts and ideas in mind, we would like to offer a somewhat provocative view that perhaps plasmids, megaplasmids, and chromids should be considered as a spectrum that varies in multiple characteristics including size and ‘essentiality’ rather than generic placement into defined orthogonal groups. There exist relatively small plasmids that are absolutely essential for physiological functioning of cells, such as the 9.4 kb ribosomal RNA-encoding plasmids of *Aureimonas* [48], and gigantic plasmids that are expendable and whose fitness effects are only apparent in specific niches and contexts, for example, the 1.35 Mb pSymA of *Sinorhizobium meliloti*, deletion of which has only minor effects on growth, transcription and proteome phenotypes [49–51]. Some small plasmids exist entirely as mosaics from multiple contributing replicons while some large plasmids have remained syntenic despite extensive divergence in sequence [52,53]. If one were to reconceptualize plasmid terminology, taken together, perhaps it would be best to simply discuss the characteristics of each replicon itself rather than worry about nomenclatural challenges. After all, just like a vacuum, nature often abhors cleanly delineated conceptual taxonomic schemes. We, therefore, caution against absolutes in determining what counts as a megaplasmid.

**Figure 3.**
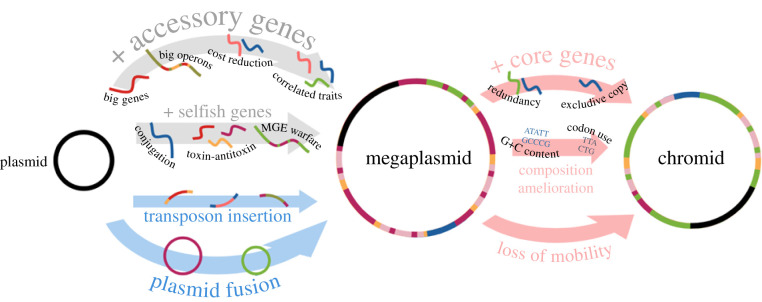
What makes a megaplasmid. Grey arrows; megaplasmids arise and persist when plasmids are selected to carry more accessory gene content: big genes or operons, multiple different environmentally correlated traits, and/or genes that reduce physiological burdens. At the same time, ‘selfish’ traits that promote plasmid vertical and horizontal transmission can also drive up plasmid size. Blue arrows; new genes can be acquired by the activities of other mobile genetic elements, including transposons, plasmids, ICEs and phage (not shown). Red arrows; chromids are thought to develop from megaplasmids by acquiring core genes, potentially through an intermediate step of carrying a redundant copy. Chromids also tend to lose the ability for horizontal transmission. Consequently, ‘locked in’ to a particular genome, the nascent chromid acquires the compositional signatures of the host chromosome.

An analogy can perhaps be found in the use of the term ‘megafauna’ by conservation biologists to refer to animals larger than some threshold mass, a threshold that, as with megaplasmids, varies across studies and the type of organism in question [54]. Different megafauna species may be more closely related to smaller animals than they are to one another, for example, an elephant is more similar genetically to the pocket-sized golden mole than to the bison. However, similar evolutionary pressures are probably responsible for the large size of both bison and elephants, and genetically diverse megafaunal species can play similar ecological roles. We suggest that, just as ‘megafauna’ remains a useful term for conservation ecologists, ‘megaplasmid’ can remain a useful term to define plasmids that are big relative to the genomes they come from, even if megaplasmids cannot be considered a coherent group in terms of an unambiguous size threshold, evolutionary relatedness, or specific gene content. A loose term, like megaplasmid or megafauna, encourages us to generalize to understand the underlying biology. In the next sections, we discuss these features of megaplasmids—their size, genetics, and gene content—that explain their evolutionary and ecological role (figure 3).

## 3. Why are megaplasmids bigger?

Selection simultaneously acts on plasmids from multiple levels [55]. Selection acts on plasmids directly, favouring those that are more effective at persisting and transmitting. At the same time, selection acts at higher levels of biological organization, for example, at the level of the cell or the population, indirectly favouring plasmids that enhance the fitness of their microbial hosts. Selection also acts at lower organizational levels: that of transposons or insertion sequences, which can exploit plasmids to spread to new hosts. Plasmid gene content is shaped by these different selective pressures (alongside genetic drift, which is thought to be relatively weak in most microbial populations [56]). Selection at different organizational levels is not necessarily aligned—in fact, it is often assumed that there is some trade-off between host-level and plasmid-level selection, such that plasmids which are more effective at transmission are more of a burden on their hosts, while those that benefit their hosts become less proficient at horizontal transmission [57,58]. In the long run, plasmids might be expected to become either efficient parasites that have shed any genetic cargo that does not enhance their selfish reproduction, or domesticated mutualists that are incapable of autonomous replication or transmission [59]. Prokaryotic genomes are subject to a deletional bias, and thus plasmids exist under a constant threat of gene loss [60]. It may therefore seem surprising to find large megaplasmids that both confer many beneficial traits, and are capable (and even highly proficient) at spreading to new hosts: a case in point is the approximately 400 kb pBT2436-like family of *Pseudomonas* megaplasmids, which transmit multiple resistance determinants at a very high rate [24]. In fact, there are many ways in which megaplasmids might be successful because of, not despite, their large size.

### (a) Larger plasmids can be more effective parasites

Expanding gene content is one way in which larger plasmids can be more efficiently inherited (i.e. vertical transmission), and more efficiently spread (i.e. horizontal transmission). Vertical transmission requires viable offspring cells to receive a copy of the plasmid. Partitioning systems, which mechanically distribute copies of the plasmid to offspring [61], are encoded by low copy-number (approx. less than 5) plasmids (such as large plasmids tend to be [62]) out of necessity because the risks of loss by segregation are untenable [63]. However, such systems are imperfect, and if plasmid-free cells do not suffer the burdens of carriage, the plasmid can be driven out of the population by purifying selection. Therefore, larger plasmids tend to also carry toxin-antitoxin (TA) systems which ensure that plasmid-free cells do not succeed [64]. These linked genes, encoding a long-lived toxin and a short-lived antitoxin, result in an (often lethal) fitness cost if the antitoxin gene is lost [65], and are often found to be necessary for megaplasmid stability, as was seen for pTTS12 (583 kb) and pAtC58 (542 kb) [66,67]. In fact, evolution experiments selecting for increased stability of a 43 kb IncP-1beta plasmid found this plasmid reproducibly acquired a TA system from the chromosome, enhancing its persistence and increasing its size [68,69]. Some plasmids encode many different TA systems, with as many as seven detected on the 103 kb *Synechocystis* plasmid pSYSA [70]. TA systems are themselves relatively small, meaning that the genes themselves can make only a negligible contribution to plasmid size, but several TA systems distributed across a plasmid can stabilize larger regions that would otherwise be prone to deletion [71,72].

Horizontal transmission of plasmids is thought to occur mainly by conjugation [73,74]; this is particularly the case for larger plasmids which are likely to exceed the packaging limits of most phage capsids, thus preventing efficient transduction (though see [75]), and are more vulnerable to extracellular damage, thus inhibiting transformation [76]. Conjugation requires a conjugative pilus: a macromolecular structure comprising approximately 12 proteins or more [77,78], and thus the genetic size of the transfer region means that self-conjugative plasmids tend to be large [33]. Many megaplasmids are predicted to encode conjugative machinery, with experiments showing conjugation sometimes at very high rates [24,79–81]. Though rare [13,82], some plasmids carry multiple conjugative and replication machineries (e.g. [79]), potentially extending the range of conditions in which they can transfer and hosts in which they can be maintained, with bigger plasmids more likely to carry multiple replication modules (electronic supplementary material, figure S4). Horizontal transmission can also be enhanced by plasmid-encoded pili and fimbriae that promote biofilm formation and cell-cell adhesion [83,84]. However, though larger plasmids are more likely to be conjugative (electronic supplementary material, figure S3), some megaplasmids appear to lack conjugative machinery altogether, and either depend on the conjugative machinery of other plasmids to transmit (i.e. they are mobilizable) or are apparently not mobile at all [85]. Annotation algorithms that use the well-described conjugative machineries of *Enterobacteriaceae* as templates may underperform when detecting transfer genes in non-conventional organisms [86], and the thick seam of uncharacterized megaplasmid genes may yet show new mechanisms for horizontal transmission. Alternatively, non-mobilizable megaplasmids may be *en route* to becoming chromids, as discussed below.

Plasmids occupy a world inhabited by other plasmids. Interacting mobile genetic elements can exploit one another's transmission machineries, compete for cellular resources, and directly exclude one another (for example, plasmids of the same incompatibility group) [87,88]. Co-infection of a prokaryote with multiple different plasmids is common [89], and mechanisms have evolved to promote one plasmid over another. Some plasmids pack a punch by carrying their own CRISPR-Cas systems, to eliminate other plasmids [53,90,91]. CRISPR-encoding plasmids tend to be conjugative and relatively big (the CRISPR locus itself is approx. 6 kb [91]), and interestingly, the targets of these ‘plasmid-hunting plasmids' tend also to be relatively large, suggesting that CRISPR-mediated plasmid warfare tends to be a clash of titans [90,92].

### (b) Larger plasmids can be more effective mutualists

Many plasmids carry ‘accessory genes’: genes that do not confer a direct fitness benefit on plasmids, and instead benefit plasmids indirectly by improving the fitness of the microbes that carry them. Accessory genes confer functions as diverse as virulence, antibiotic resistance, metabolism, biofilm production, and gene regulation [83,93–95]. Plasmids that provide beneficial accessory genes have fitness interests aligned with those of their hosts—where hosts benefit, the plasmid benefits too. Under such circumstances, selection and drift would be expected to eventually rob plasmids of their autonomy, through the loss of horizontal transmission, and/or integration of beneficial accessory genes into the chromosome. However, beneficial plasmids persist [59,96]. A powerful explanation is the ‘local adaptation’ model of plasmid accessory gene content [55,97]. Briefly, the local adaptation model states that some traits are beneficial—or indeed, may be essential—only in certain specific situations or environments. New bacterial strains colonizing that environment are at a disadvantage compared with the incumbent if they do not have the trait; this will be the case even if the incoming strain has beneficial mutations at other positions in the genome. A plasmid that can transmit locally-adaptive traits into a competitive newcomer enables that newcomer to succeed, indirectly benefiting the transmissible, accessory-gene-encoding plasmids in the process [96,98].

The local adaptation model can explain why some plasmids are so large because in some cases, the genes involved are themselves very big. For example, the three megaplasmid-borne polyketide synthase genes MLSA1, MLSA2, MLSB, which enable *Mycobacterium ulcerans* to produce mycolactone toxin and thus cause infection, are 51 kb, 7 kb and 42 kb respectively, forming a locus that exceeds 100 kb in size [99]. While such giant genes are an exception, large gene clusters, encoding a series of enzymes all necessary for biosynthesis, catabolism, or virulence, are often found on megaplasmids: the nicotine catabolism pathway of the *Arthrobacter nicotinovorans* pAO1 [100] and the entry region of the *Shigella* virulence plasmid [93] provide just two examples.

Larger plasmids can carry many different locally adaptive genes. But in other cases, traits are encoded on different, coexisting replicons [101]. What might cause locally-adaptive traits to accumulate on a single large plasmid, rather than being distributed across multiple smaller plasmids? The ‘selfish operon’ hypothesis proposes that a group of accessory genes which contributes to a single beneficial function becomes linked or clustered in bacterial genomes, because each gene benefits from being co-transferred [102]. In other words, gaining the genes for this function separately provides no benefit until the whole set has been acquired. Genes that are clustered—and therefore probably transfer together—are more likely to provide recipients with the full set, and hence the corresponding fitness benefits. The argument can be extended in the light of the local adaptation model to explain multi-trait plasmids. Here, we can define the ‘function’ of the accessory genes as adaptation to a single specific environment, for example, an environment contaminated with several toxic substances. Lacking resistance to just one toxin might be lethal, and so locally adaptive plasmids will be most successful if they confer resistance to all. At a broader scale, this dynamic would play out to shape plasmid accessory gene content: where there is correlation between different local selective pressures, there is selection for the corresponding adaptive traits to become aggregated on a single mobile plasmid [98]. Where there are many of these locally correlated environmental pressures, we might expect to see larger plasmids.

There are several examples of multi-trait plasmids that may reflect these pressures. The use of antibiotics in clinical and agricultural settings means that microbes inhabiting those environments often face several specific chemical threats in contrast to closely-related strains in the wider environment. Large resistance plasmids often carry mechanisms to resist not only many different antibiotics (electronic supplementary material, figure S2), but also disinfectants, and metals, occasionally or historically used as antiseptics [103]. Toxic metals often co-occur in industrial waste from factories and mining, and similarly selects for plasmids with resistance to multiple metals/metalloids [104]. In soil, microbes can exploit diverse, chemically related, but low-abundant compounds produced by plants, thanks to multifunctional catabolic plasmids [94,105], which may explain why megaplasmids are more common in families such as Rhizobiaceae, Burkholderiaceae and Pseudomonadaceae, which often associated with soil (figure 2). Genes enabling nitrogen fixation and hydrogenotrophy are often conferred by the same plasmid because H_2_ is produced during the reduction of dinitrogen to ammonia, and hydrogenotrophy enables microbes to recover some of the energetic costs of nitrogen fixation [106]. Where adaptation to a new environment requires the concurrent expression of several different traits, larger plasmids can provide.

However, the functional traits encoded by megaplasmids are often only a small portion of the whole sequence. Many megaplasmid genes are of unknown function, and it is not clear whether these are selfish or mutualistic. Much work is required to characterize their biochemical and evolutionary roles, but recent work looking for genes that co-localize with known anti-phage mechanisms has enabled several new genome defence pathways to be identified [107]. Perhaps similar approaches with locally-adapted plasmids may shed light on the ‘known unknowns’ of the prokaryote pangenome.

Besides accessory gene functions, megaplasmids can become more effective mutualists by reducing the impact they have on the physiology of their hosts. Some plasmids come equipped with transfer RNAs (tRNAs), which appear to be more common on larger plasmids (electronic supplementary material, figure S5), and along with other translation- and transcription-related genes may alleviate the metabolic burdens associated with carriage [53], as has been shown for tRNA-encoding prophage [108]. Plasmid burden can also be ameliorated by nucleoid-associated proteins like histone-like nucleoid structuring, which are often found on larger plasmids and can regulate plasmid gene expression by bridging distant DNA sequences to shape nucleoid architecture [109]. Anti-SOS proteins likewise minimize plasmid-associated disruptions [110]. Furthermore, megaplasmids, and their prokaryotic hosts, can evolve and coevolve to minimize burden. Single mutations can largely ameliorate carriage costs of some megaplasmids, and laboratory studies show that at least under certain conditions, some megaplasmids have no detectable fitness cost, suggesting that size *per se* is not principally responsible for megaplasmid fitness costs [111]. Very big plasmids are not necessarily very burdensome plasmids.

Continued positive selection for plasmid cargo genes means that plasmids can remain associated with a chromosome for a long time. Under these circumstances, adaptive and neutral processes favour an increasingly refined relationship between plasmid and chromosome, a process of plasmid 'domestication', the results of which are most clearly seen as chromids.

## 4. Are megaplasmids just chromids-in-training?

Most chromids characterized to date are relatively big, with replication machinery like that of plasmids, and it is likely that megaplasmids are the evolutionary progenitors of chromids [19,21]. Indeed, chromids often appear in plasmid databases and are sometimes considered as a form of large plasmid [13]. Two key features distinguish megaplasmids from chromids. First, chromids tend to more closely resemble their associated chromosomes in terms of nucleotide composition, suggestive of a prolonged association [19,21]. Consistent with this, chromids appear not to be horizontally transmitted, at least under natural conditions, and are instead inherited vertically [19,21]. Loss of horizontal transmission has been reported in evolution experiments with large conjugative plasmids, indicating the ease with which megaplasmids can become exclusively associated with a lineage, beginning the domestication process [112,113]. The other key distinction is that chromids encode core cellular functions, that in other species are on the chromosome, the implication being that cells cured of their chromids are not viable under any condition [21]. On a global level, core genes are less likely to undergo successful horizontal gene transfer, owing to the difficulties associated with integrating a foreign gene variant into the tight-knit protein interaction networks characteristic of core cellular activity [114]. However, like chromids, mobile megaplasmids also occasionally carry homologues of core genes. These genes may have distinct functions, but in some cases, such as the metabolic genes present on the resident extrachromosomal replicons of *Sinorhizobium meliloti*, or plasmid-borne chaparonin genes found in *Escherichia*, have been shown to complement mutations in their cognate chromosomal copies [115,116], indicative of genetic functional redundancy [117].

Redundant plasmid-borne core genes can play an important role in evolution. The loss of one copy of a redundant function, either through drift, or by selection (also known as Black Queen dynamics), renders the remaining copy essential, increasing interdependency between partners [118]. In a genome with both plasmid and chromosomal copies of a core gene, disruption of the chromosomal copy may have no phenotypic effect, but immediately renders the plasmid essential, dividing (albeit asymmetrically) core cellular functions across two replicons [40]. This increased genomic complexity may not have any immediate functional or mechanistic adaptive benefit [119], but it establishes the plasmid in the genome, exposing it to local mutational biases, and enabling subsequent evolutionary processes to exploit the gene regulatory or replication opportunities offered by a multipartite genome. Redundancy also enables core cellular functions to accumulate deleterious mutations, shielded from the purifying selection such genes would experience were they single-copy [120]. Plasmids thus act as ‘scribbling pads’ [121], with redundant genes and non-adaptive features providing a source of standing genetic variation that can enable evolution to traverse valleys in the fitness landscape [32].

The presence of redundant core genes on megaplasmids has inspired a model whereby megaplasmids are destined to either evolve into a chromid, or be lost [19]. However, distantly-related conjugative megaplasmids show clear gene synteny despite nucleotide sequence divergence, suggesting that these families have persisted and diversified as large, horizontally transmissible mobile genetic elements [24,25,53,80]. Some megaplasmids have clearly been around for a long time, suggesting that they have found success in a particular niche of the sub-cellular ecosystem, rather than representing a transitional state on the way to domestication.

## 5. Plasmid plasticity promotes megaplasmid emergence

While some megaplasmids seem to be ancient, the plasticity of plasmids provides many opportunities for new megaplasmids to arise [46]. There are many cases where large plasmids are clearly fusions, or mosaics, of two or more smaller replicons [43]. For example, in *Bacillus cereus*, a study found 29 out of 31 plasmids greater than 100 kb carried multiple different replication machineries, with phylogenetic analyses showing different evolutionary histories within each megaplasmid [122]. A megaplasmid in *Klebsiella pneumoniae* provides a concerning case where virulence and antimicrobial resistance (AMR) functions, which usually have distinct patterns of horizontal transmission, became conjoined, with unclear implications for public health [123,124]. Such fused megaplasmids may not be long-lived, but nevertheless provide powerful new evolutionary opportunities.

Plasmids can usually accumulate transposons more easily than chromosomal loci, because the genes they carry are not essential. As the transposon itself is redundant, it then provides a site for successive transposons to insert with negligible disruption. This generates 'hotspot' or 'junkyard' regions, which can accumulate new insertions, and thus standing genetic variation [125]. Such hotspot regions could also accelerate plasmid expansion, pushing large plasmids over the threshold into becoming megaplasmids. Transposons can also interact with one another or with their target sites, causing further rearrangements [126]. For example, homologous recombination between different copies of the same transposon can cause the capture of plasmid genes to the chromosome [127], and genes from other replicons to be acquired by plasmids [128], potentially mobilizing those genes to transmit onwards into new recipients. Conversely, interactions between transposons can also cause gene deletion from megaplasmids [113,129]. In many ways, the large size of megaplasmids reflects the plasticity of large plasmids more broadly, exemplifying the ease with which they can acquire and shuttle constituents of the pangenome.

## 6. Boxes: megaplasmids to watch

### (a) *Salmonella enterica* infantis, an emerging pathogen with an emerging megaplasmid: pESI (280 kb)

*Salmonella enterica* serovar Infantis (*S.* Infantis) is a globally emerging serovar of *Salmonella enterica* and a common cause of human salmonellosis [130]. The dominant lineage, which has become established in poultry and now poses most risk to humans owes its success to a virulence-resistance megaplasmid (approx. 280 kb) known as pESI [130–133]. pESI-like megaplasmids have now been found in *S*. Infantis isolates from around the world [134]. pESI confers several functions associated with pathogen success in environments of high antimicrobial usage: resistance to diverse antimicrobials, including mercury and oxidative stress, a siderophore cluster for iron acquisition during infection, and fimbriae genes, which might enable biofilm formation and epithelial adhesion [132]. Overall, the content of the megaplasmid varies between isolates, consistent with a mosaic structure that is derived from both IncI1 and IncP-1alpha plasmids, as well as different transposons [132,135]. pESI appears to have been principally inherited vertically by the emerging *S.* Infantis lineages, but there is evidence that it transmits readily between gut commensals and other pathogens [135].

### (b) *Pseudomonas* antimicrobial resistance megaplasmids, bridging the environment and the clinic: pBT2436 (423 kb) and relatives

Incompatibility typing and gel electrophoresis in the 1970s and 1980s revealed a large group of *Pseudomonas* megaplasmids (approx. 500 kb) known as IncP-2, which, in nosocomial isolates, carried antimicrobial resistance, and in soil isolates carried pathways for degradation of complex organic compounds such as camphor and octane [136]. Spurred on by the availability of long-read sequencing—often necessary to resolve their complex or repetitive structures [24,26]—recent years have seen an increase in the number of related *Pseudomonas* megaplasmid sequences [24,25,79,137], challenging the prevailing viewpoint that plasmids make only a minor contribution to antimicrobial resistance in *Pseudomonas* [138]. Found in clinical samples, soil isolates, and industrial processes, this family exemplifies the mosaic nature of megaplasmids and their ability to confer locally-adaptive traits. The conserved, syntenic region putatively encodes a metabolic gene cluster, chemotaxis apparatus, and a type IV pilus, as well as genes involved in plasmid replication, segregation, and transmission, but members of this family also possess a variable region with sample-specific gene content [24,26,139]. The apparently high stability, low fitness cost, and efficient transmission of these megaplasmids suggests that they could be effective vehicles of gene exchange in diverse habitats.

### (c) *Acinetobacter* megaplasmids, global distribution, local adaptation: pR4WN-1BD1 (285 kb) and relatives

Interestingly, a pattern similar to that of *Pseudomonas* AMR megaplasmids is emerging in another environmentally-widespread bacterium and occasional opportunistic pathogen: *Acinetobacter* spp. Acinetobacter plasmids are often big: 10% in NCBI GenBank were found to be greater than 100 kb [140]. A combination of PacBio and HiSeq sequencing recently revealed a family of megaplasmids (250–400 kb in size) transmitting multi-drug resistance across 11 different *Acinetobacter* species. A role for local adaptation in shaping plasmid accessory gene content was discernible in these data, as plasmids from similar geographical locations had similar accessory genes, even if they were not closely related [27]. Increased sampling of non-clinical sites may be necessary to reveal the full megaplasmid picture in *Acinetobacter*, as an analysis of *Acinetobacter* plasmids from hospitals and soil/water suggested that larger plasmids were obtained from environmental samples [141].

### (d) Haloarchaeal megaplasmids, gas vesicle giants

While plasmids have been identified across Archaea, large plasmids and megaplasmids seem more restricted, having been reported only in Haloarchaea and in some species of Methanosarcinales [14]. However, amongst the Haloarchaea, megaplasmids (greater than 130 kb) are common [142]. Recombination between plasmids and chromosomes occurs frequently, probably facilitated by the transposable elements often present at very high density on haloarchaeal megaplasmids [143]. As a consequence of this dynamic genome architecture, chromosomal genes are often found on haloarchaeal megaplasmids, sometimes resulting in the megaplasmid becoming an essential chromid [144]. Haloarchaeal megaplasmids are distinct amongst the mainly parasitic Archaeal extrachromosomal elements because they confer known accessory traits: in particular, gas vesicles that assist with buoyancy [14,144]. Some haloarchaeal megaplasmids do encode genes resembling bacterial conjugative apparatus [144], but horizontal gene transfer between Haloarchaea readily occurs via cell fusions which can transmit chromosomal as well as plasmid DNA [145]. The origin of the haloarchaeal megaplasmids has attracted speculation because megaplasmids are uncommon in Archaea. This has lead some to suggest that modern haloarchaeal megaplasmids emerged from an ancient inter-kingdom transmission event [14], which, considering the ease with which Haloarchaea form cytoplasmic bridges with their neighbours, along with phylogenetic analyses which indicate that the common ancestor of all Haloarchaea acquired approximately 1000 genes from Eubacteria [146], is a compelling hypothesis.

## 7. Conclusion and perspective

Megaplasmids are not the only maxi-size mobile genetic element to rise to prominence with new sequencing efforts. Large symbiosis ICEs (greater than 400 kb) are frequently spotted on *Mesorhizobium* spp. [147,148]. Huge bacteriophages, also known as jumbophage, have been identified and sequenced from isolates and from metagenomes, with the largest to date at 735 kb [149]. As with megaplasmids, many jumbophage genes are of unknown function, but some functions have been identified: CRISPR systems, nucleoid structuring proteins, and genes that support and manipulate host translation during the infection process [149,150]. ‘Borgs’—linear megaplasmids up to 1 Mbp in size—appear to confer locally-adaptive ecologically-significant functions to the methane-oxidising Archaeon, *Methanoperedens* [151]. The size and complexity of large mobile genetic elements demonstrates that sub-cellular life need not lack sophistication when undertaking large-scale reprogramming of cellular functions. As more—possibly even larger—elements are discovered through continued, broader, less-biased sequencing initiatives, we will develop an increasingly detailed picture of the intriguing ecologies and adaptations of huge mobile genetic elements.

From their original isolation and detection with modified DNA extraction techniques, to pulse-field gel analysis, to a renaissance facilitated by long-read sequencing, knowledge of megaplasmids in particular, and multipartite genome structure in general, has advanced with each technological development. What comes next? We anticipate that wider genomics and metagenomics surveys, informed by long-read sequencing, will expand our understanding of the habitat distribution of megaplasmids and chromids, as well as their accessory gene content. However, experiments, including laboratory evolution and detailed molecular and structural studies, will be crucial to test megaplasmid biochemical, ecological, and evolutionary functions. Contact sequencing approaches such as Hi-C and meta3C will provide powerful insight into the spatial structure of mobile genetic elements both within cells and within communities, enabling an understanding of how plasmids and chromids interact with chromosomal architecture, and population dynamics and drivers of transmission [152–154]. Finally, pangenomics analysis pipelines, combined with the ever-increasing abundance of sequencing data, will improve our knowledge of megaplasmid cargo, and how megaplasmids co-associate (or anti-associate) with other components of the accessory genome within cells [155]. Such work, alongside comparisons with other mobile genetic elements and routes of gene exchange, will help contextualize the role played by these high-capacity vehicles for horizontal gene transfer, giants of the sub-cellular world.

## Data accessibility

Data presented in this manuscript are already publicly available through accessible databases (NCBI, COMPASS, EZBioCloud). These databases are referenced in the text, and description is provided in the text for how they were accessed.
